# Supplementary material for: A harm reduction model for environmental tobacco smoke exposure among Bangladeshi rural household children: A modified Delphi technique approach
Source: PLoS One. 2023 Feb 16;18(2):e0276424. doi: 10.1371/journal.pone.0276424 (PMC9934442; doi:10.1371/journal.pone.0276424)
Supplement: S1 File — (PDF) [file pone.0276424.s001.pdf]

QUESTIONNAIRE

QUESTIONNAIRE ID NUMBER:	
UPAZILLA (SUB DISTRICT):	
UNION/WARD:	
VILLAGE/ MOUZA /MOHALLAH:	

INSTRUCTIONS:

- PLEASE READ EACH QUESTION CAREFULLY BEFORE ANSWERING IT.
- CHOOSE THE ANSWER THAT BEST DESCRIBES WHAT YOU BELIEVE AND FEEL TO BE CORRECT.
- CHOOSE ONLY ONE ANSWER FOR EACH QUESTION.
- ON THE ANSWER SHEET TICK (✔) THE SQUARE BOX (□) THAT CORRESPONDS TO YOUR ANSWER

A. BACKGROUND CHARACTERISTICS

A1. YOU OR ANYONE ELSE SMOKE TOBACCO IN YOUR HOUSEHOLD?

- □ 0. NO
- □ 1. Yes

A2. ARE YOU A…………?

- □ 1. FATHER
- □ 2. MOTHER
- □ 3. SON
- □ 4. DAUGHTER
- □ 5. OTHERS (SPECIFY)_______________

A3. WHAT IS YOUR GENDER?

- □ 1. MALE
- □ 2. FEMALE

A4. WHAT IS YOUR AGE?

____

A5. WHAT IS YOUR MARITAL STATUS?

- □ 1. SINGLE
- □ 2. MARRIED
- □ 3. DIVORCE
- □ 4. WIDOW
- □ 5. OTHERS (SPECIFY)_______________

A6. WHAT IS THE HIGHEST LEVEL OF EDUCATION YOU HAVE COMPLETED?

- □ 1. NO FORMAL SCHOOLING

- ☐ 2. PRIMARY SCHOOL
- ☐ 3. SECONDARY SCHOOL
- ☐ 4. COLLEGE COMPLETED
- ☐ 5. UNIVERSITY COMPLETED

A7. WHAT IS YOUR RELIGIOUS BELIEF?

- ☐ 1. ISLAM
- ☐ 2. HINDU
- ☐ 3. CHRISTIAN
- ☐ 4. BUDDHIST
- ☐ 5. OTHERS (SPECIFY) _______________

A8. WHAT IS YOUR MAIN OCCUPATION?

- ☐ 1. SERVICE HOLDER
- ☐ 2. BUSINESS
- ☐ 3. AGRICULTURAL WORKER/ DAY LABOUR
- ☐ 4. HOUSEWIFE
- ☐ 5. STUDENT
- ☐ 6. UNEMPLOYED

A9. WHAT IS YOUR MONTHLY FAMILY INCOME?

- ☐ BDT_______________

A10. HOW MANY CHILDREN DO YOU HAVE?

- ☐ _______________

A11. WHAT ARE THE AGE OF YOUR CHILDREN (IF A1 IS YES)?

- ☐ _______________

B. PASSIVE SMOKING EXPOSURE:

B1. ARE YOU EXPOSE TO PASSIVE SMOKING (EXPOSURE OF PASSIVE SMOKING INDICATES AS EXPOSURE TO ANOTHER PERSON'S TOBACCO SMOKE IN THE HOUSEHOLD FOR AT LEAST 15 MINUTES DAILY FOR MORE THAN ONE DAY EVERY WEEK IN THE PAST 30 DAYS)

- ☐ 1. YES
- ☐ 0. NO

C. SMOKING INFORMATION (IF YOU ARE A SMOKER):

C1. WHAT IS YOUR SMOKING STATUS?

- ☐ 1. SMOKER
- ☐ 2. NON-SMOKER

C2. IS THERE ANYONE ELSE SMOKE TOBACCO INSIDE YOUR HOUSE?

- ☐ 1. YES
- ☐ 0. NO

C3. DO YOU CURRENTLY SMOKE TOBACCO?

- 1. DAILY
- 2 LESS THAN DAILY
- 3. NOT AT ALL

C4. AT WHAT AGE YOU FIRST STARTED SMOKING TOBACCO DAILY?

- 1. __________ YEARS OLD

C5. WHAT FORM OF TOBACCO YOU SMOKE EVERYDAY?

- 1. MANUFACTURED CIGARETTES?
- 2. BIDIS?
- 3. OTHER (SPECIFY)__________________

C6. HOW MANY ROLL YOU SMOKE EVERYDAY IN LAST 30 DAYS?

- ________________

C7. FROM WHERE YOU USUALLY BUY SMOKING TOBACCO PRODUCT IN LAST 30 DAYS?

- 1. LOCAL SHOP
- 2. STREET VENDOR
- 3. SUPERMARKET

C8. WERE THESE CIGARETTES FILTERED OR NON-FILTERED?

- 1. FILTERED
- 2. NON-FILTERED

C9. HOW MAY ROLL YOU USUALLY BUY AT A TIME IN LAST 30 DAYS?

- ________________ ROLLS

C10. HOW MUCH YOU EXPANSE FOR BUYING TOBACCO PRODUCT EVERY MONTH?

- BDT _______________

D. HOUSEHOLD RULES ABOUT SMOKING

D1. IS SMOKING ALLOWED INSIDE YOUR HOME?

- 1. YES
- 0. NO

D2. INSIDE YOUR HOME, IS SMOKING ALLOWED IN EVERY ROOM?

- 1. YES
- 0. NO

D3. HOW OFTEN DOES ANYONE SMOKE INSIDE YOUR HOME?

- 1. DAILY
- 2. WEEKLY
- 3. MONTHLY
- 4. NEVER

D4. DO YOU OR OTHER SMOKE IN FRONT OF A CHILD INSIDE HOUSE?

- ☐ 1. YES
- ☐ 0. NO

D5. DO YOU BUY SMOKING PRODUCT BY YOUR CHILD?

- ☐ 1. YES
- ☐ 0. NO

D6. WHO MADE THE RULES ABOUT SMOKING INSIDE HOUSE?

- ☐ 1. FATHER
- ☐ 2. MOTHER.
- ☐ 3. GRANDPARENTS
- ☐ 4. NO ONE SPECIFICALLY

E. MEDIA:

E1. DID YOU SEE/HEAR ANY ANTI-TOBACCO ADVERTISEMENT WITHIN LAST 30 DAYS?

- ☐ 1. YES
- ☐ 0. NO

E2. WHERE DID YOU SEE/HEAR THE ANTI-TOBACCO ADVERTISEMENT (IF E1 IS YES)?

- ☐ 1.NEWSPAPER
- ☐ 2.MAGAZINE
- ☐ 3.POSTER
- ☐ 4.RADIO
- ☐ 5.TELEVISION
- ☐ 6. INTERNET
- ☐ 7. INSIDE SHOP

E3. DID YOU SEE/HEAR ANY TOBACCO ADVERTISEMENT WITHIN LAST 30 DAYS?

- ☐ 1. YES
- ☐ 0. NO

E4. WHERE DID YOU SEE/HEAR THE TOBACCO ADVERTISEMENT IF E3 IS YES?

- ☐ 1.NEWSPAPER
- ☐ 2.MAGAZINE
- ☐ 3.POSTER
- ☐ 4.RADIO
- ☐ 5.TELEVISION
- ☐ 6. INTERNET
- ☐ 7. INSIDE SHOP

E5	DO YOU THINK MEDIA CAN HELP TO REDUCE SMOKING?	☐ 1. YES	☐ 0. NO
E6	DOES ANY ADVERTISE MAKE YOU THINK ABOUT USING TOBACCO?	☐ 1. YES	☐ 0. NO
E7	DID YOU RECEIVE ANY FREE CIGARETTE FROM ANY COMPANY/SHOP IN THE LAST 6 MONTHS?	☐ 1. YES	☐ 0. NO
E8	DID YOU RECEIVE ANY GIFT (EG. SHIRT, PEN OR ANY OTHER THING) FROM ANY COMPANY/ SHOP IN THE LAST 6 MONTHS?	☐ 1. YES	☐ 0. NO

F. SOCIAL NORM AND CULTURE

F1	WHAT DO YOU THINK 'NOT TO SMOKE IN FRONT OF ELDER HELP REDUCE EXPOSURE?	☐ 1. AGREE	☐ 0. NOT AGREE
F2	WHAT DO YOU THINK IF THERE IS NO BARRIER TO ASK HUSBAND NOT TO SMOKE CAN REDUCE EXPOSURE?	☐ 1. AGREE	☐ 0. NOT AGREE
F3	DO YOU THINK SOCIAL SMOKING INCREASES EXPOSURE OF PASSIVE SMOKING?	☐ 1. AGREE	☐ 0. NOT AGREE
F4	DO YOU THINK CEREMONIAL SMOKING INCREASES PASSIVE SMOKING EXPOSURE?	☐ 1. AGREE	☐ 0. NOT AGREE

G. HOUSEHOLD ENVIRONMENT:

G1. HOW MANY ROOMS IN YOUR HOUSE?

- ☐ 1. ONE
- ☐ 2. MORE THAN ONE

G2. WHAT TYPE OF HOUSE YOU STAY?

- ☐ 1. BUILDING
- ☐ 2. TIN
- ☐ 3. FENCE

G3	DO YOU HAVE OPEN YARD?	☐ 1. YES	☐ 0. NO
G4	IS YOUR HOUSE NEAR TO ANY SHOP WHERE CIGARETTE IS SOLD?	☐ 1. YES	☐ 0. NO
G5	DOES ANY CHILD SLEEP WITH YOU?	☐ 1. YES	☐ 0. NO
G6	DOES ALL THE ROOM HAVE WINDOWS?	☐ 1. YES	☐ 0. NO

H. KNOWLEDGE:

H1	TOBACCO SMOKE CAUSE HARM TO A CHILD	☐ 1. TRUE	☐ 0. FALSE
H2	IF A CHILD IS HEALTHY TOBACCO SMOKE DOES NOT HAVE ANY AFFECT	☐ 1. TRUE	☐ 0. FALSE
H3	TOBACCO SMOKE EXPOSURE CAUSES RESPIRATORY PROBLEM OF CHILDREN	☐ 1. TRUE	☐ 0. FALSE
H4	TOBACCO SMOKE EXPOSURE CAUSES CANCER	☐ 1. TRUE	☐ 0. FALSE
H5	TOBACCO SMOKE EXPOSURE CAUSES ASTHMA	☐ 1. TRUE	☐ 0. FALSE
H6	TOBACCO SMOKING HAS ONLY EFFECT ON SICK CHILDREN	☐ 1. TRUE	☐ 0. FALSE
H7	LITTLE EXPOSURE HAS NO HARM TO A CHILD	☐ 1. TRUE	☐ 0. FALSE
H8	PUBLIC SMOKING IS BAN IN BANGLADESH	☐ 1. TRUE	☐ 0. FALSE

I. PERCEPTION:

I1	I BELIEF THAT PASSIVE SMOKING IS HARMFUL FOR CHILDREN	☐ 3. AGREE	☐ 2.NEITHER AGREE OR DIS AGREE	☐ 1.DISAGREE
I2	I THINK A PARENTS CAN PROTECT THEIR CHILD FROM TOBACCO SMOKE EXPOSURE	☐ 3. AGREE	☐ 2.NEITHER AGREE OR DIS AGREE	☐ 1.DISAGREE
I3	I THINK PARENTS HAVE THE RIGHT TO DECIDE WHETHER SMOKING IS ALLOWED IN FRONT OF CHILD	☐ 3. AGREE	☐ 2.NEITHER AGREE OR DIS AGREE	☐ 1.DISAGREE

I4	I THINK THERE SHOULD HAVE LAW OF SMOKING BAN INSIDE HOME LIKE PUBLIC PLACE BANNING	☐ 3. AGREE	☐ 2.NEITHER AGREE OR DIS AGREE	☐ 1.DISAGREE
I5	I THINK IT'S PARENTS RESPONSIBILITY TO KEEP AWAY CHILDREN FROM TOBACCO SMOKING	☐ 3. AGREE	☐ 2.NEITHER AGREE OR DIS AGREE	☐ 1.DISAGREE

J. ATTITUDE:

J1	I DO NOT LIKE PEOPLE SMOKE INSIDE HOUSE	☐ 3. AGREE	☐ 2.NEITHER AGREE OR DIS AGREE	☐ 1.DISAGREE
J2	I DO NOT LIKE SMOKE IN FRONT OF CHILD	☐ 3. AGREE	☐ 2.NEITHER AGREE OR DIS AGREE	☐ 1.DISAGREE
J3	I THINK IT'S NOT A BIG DEAL TO SMOKE IN FRONT OF CHILDREN	☐ 3. AGREE	☐ 2.NEITHER AGREE OR DIS AGREE	☐ 1.DISAGREE
J4	I THINK CHILDREN SHOULD NEVER BE EXPOSED TO TOBACCO SMOKE	☐ 3. AGREE	☐ 2.NEITHER AGREE OR DIS AGREE	☐ 1.DISAGREE
J5	I THINK IT'S PARENTS RESPONSIBILITY TO TEACH A CHILD ABOUT TOBACCO SMOKING EXPOSURE	☐ 3. AGREE	☐ 2.NEITHER AGREE OR DIS AGREE	☐ 1.DISAGREE
J6	I THINK I AM CONFIDENCE OF MY ABILITY TO BECOME (OR STAY) NONSMOKERS	☐ 3. AGREE	☐ 2.NEITHER AGREE OR DIS AGREE	☐ 1.DISAGREE

K. INTENTION:

K1	I INTEND TO TELL NOT TO SMOKE INSIDE MY HOUSE.	☐ 3. AGREE	☐ 2.NEITHER AGREE OR DIS AGREE	☐ 1.DISAGREE
K2	I INTEND TO TELL NOT TO SMOKE IN FRONT OF MY CHILDREN	☐ 3. AGREE	☐ 2.NEITHER AGREE OR DIS AGREE	☐ 1.DISAGREE
K3	I INTEND THAT MY SPOUSE WILL NOT SMOKE WHILE CHILDREN PRESENT.	☐ 3. AGREE	☐ 2.NEITHER AGREE OR DIS AGREE	☐ 1.DISAGREE
K4	I INTENTD TO IMPLEMENTATION OF BANNING SMOKING INSIDE MY HOUSE	☐ 3. AGREE	☐ 2.NEITHER AGREE OR DIS AGREE	☐ 1.DISAGREE
K5	I INTEND TO SPECIFY A PLACE FOR SMOKING IN MY HOUSE	☐ 3. AGREE	☐ 2.NEITHER AGREE OR DIS AGREE	☐ 1.DISAGREE

L. SUBJECTIVE NORM/PEER SUPPORT:

L1	MY SPOUSE WILL HELP TO REDUCE THE EXPOSURE	☐ 3. AGREE	☐ 2.NEITHER AGREE OR DIS AGREE	☐ 1.DISAGREE
L2	MY PARENTS AND IN LAW WILL HELP TO REDUCE THE EXPOSURE	☐ 3. AGREE	☐ 2.NEITHER AGREE OR DIS AGREE	☐ 1.DISAGREE

L3	MY OTHER FAMILY MEMBERS WILL HELP TO REDUCE THE EXPOSURE	☐ 3. AGREE	☐ 2.NEITHER AGREE OR DIS AGREE	☐ 1.DISAGREE
L4	MY FRIENDS WILL HELP TO REDUCE THE EXPOSURE	☐ 3. AGREE	☐ 2.NEITHER AGREE OR DIS AGREE	☐ 1.DISAGREE
L5	MY CHILDREN WILL HELP TO REDUCE THE EXPOSURE	☐ 3. AGREE	☐ 2.NEITHER AGREE OR DIS AGREE	☐ 1.DISAGREE
L6	COMMUNITY HEALTH WORKERS/HOSPITAL WILL HELP TO REDUCE THE EXPOSURE	☐ 3. AGREE	☐ 2.NEITHER AGREE OR DIS AGREE	☐ 1.DISAGREE
L7	RELIGIOUS LEADER WILL HELP TO REDUCE THE EXPOSURE	☐ 3. AGREE	☐ 2.NEITHER AGREE OR DIS AGREE	☐ 1.DISAGREE
L8	POLITICAL LEADER WILL HELP TO REDUCE THE EXPOSURE	☐ 3. AGREE	☐ 2.NEITHER AGREE OR DIS AGREE	☐ 1.DISAGREE
L9	TEACHER WILL HELP TO REDUCE THE EXPOSURE	☐ 3. AGREE	☐ 2.NEITHER AGREE OR DIS AGREE	☐ 1.DISAGREE
L10	SHOPKEEPER WHO SELLS CIGARETTE WILL HELP TO REDUCE THE EXPOSURE	☐ 3. AGREE	☐ 2.NEITHER AGREE OR DIS AGREE	☐ 1.DISAGREE
L11	PEOPLE I WORK WITH WILL HELP TO REDUCE THE EXPOSURE	☐ 3. AGREE	☐ 2.NEITHER AGREE OR DIS AGREE	☐ 1.DISAGREE

M. PERCEIVED BEHAVIOR CONTROL:

M1	I AM CONFIDENT THAT I CAN HELP TO REDUCE EXPOSURE INSIDE MY HOUSE	☐ 3. AGREE	☐ 2.NEITHER AGREE OR DIS AGREE	☐ 1.DISAGREE
M3	I HAVE NO CONTROL TO REDUCE THE EXPOSURE	☐ 3. AGREE	☐ 2.NEITHER AGREE OR DIS AGREE	☐ 1.DISAGREE
M4	I CAN MOTIVATE OTHERS TO CONTRL SMOKING EXPOURE INSIDE HOUSE	☐ 3. AGREE	☐ 2.NEITHER AGREE OR DIS AGREE	☐ 1.DISAGREE
M6	IT IS IMPOSSIBE TO MOTIVATE OTHERS TO CONTRL SMOKING EXPOURE INSIDE HOUSE	☐ 3. AGREE	☐ 2.NEITHER AGREE OR DIS AGREE	☐ 1.DISAGREE

N. RELIGIOUS BELIEF:

N1	DO YOU THINK RELIGIOUS BELIEF CAN REDUCE THE EXPOSURE?	☐ 1. AGREE	☐ 0. NOT AGREE
N2	DO YOU THING SMOKING IS A SIN?	☐ 1. AGREE	☐ 0. NOT AGREE
N3	DO YOU THING RELIGIOUS LEADER CAN HELP PEOPLE TO REDUCE SMOKING EXPOSER?	☐ 1. AGREE	☐ 0. NOT AGREE
N4	READING RELIGIOUS BOOKS, WATCH/ LISTEN TO RELIGIOUS PROGRAMS CAN HELP TO REDUCE EXPOSURE?	☐ 1. AGREE	☐ 0. NOT AGREE

O. SOCIAL AWARNESS:

O1	INCREASES SOCIAL AWARENESS THROUGH SEMINAR	□ 1. YES	□ 0. NO
O2	INCREASES AWARENESS THROUGH LOCAL SCHOOL AMONG STUDENTS	□ 1. Yes	□ 0. No
O3	SHOPKEEPER AWARENESS	□ 1. YES	□ 0. NO
O4	PEOPLE ARE LESS AWARE ABOUT PASSIVE SMKING EXPOSURE	□ 1. Yes	□ 0. No

-----------xXXx-----------
