# Supplementary material for: A harm reduction model for environmental tobacco smoke exposure among Bangladeshi rural household children: A modified Delphi technique approach
Source: PLoS One. 2023 Feb 16;18(2):e0276424. doi: 10.1371/journal.pone.0276424 (PMC9934442; doi:10.1371/journal.pone.0276424)
Supplement: S2 File — (PDF) [file pone.0276424.s002.pdf]

Qualitative Questions for Key Informant Interview

1. What do you think about passive smoking?
2. What are the causes for passive smoking in your opinion?
3. Do you know any family who are exposed to passive smoking?
4. What do you know about passive smoking health outcome?
5. What do you do when you saw someone smoking in front of you?
6. What are the measures you will take if you want to reduce the passive smoking?
7. As a parent what you do to reduce the exposer for your children?
8. What type of family are more exposes to passive smoking in your opinion?
9. What do you know about smoking law?
10. What is your idea when someone smokes in front of your child?
11. How you can help to reduce exposer over whole?
12. What support you can give to help others for reducing exposure?

Qualitative Questions for Focus Group Discussion

1. Give your idea about passive smoking?
2. Do you think smoke free household rules can help to reduce passive smoking exposure?
3. How can we make the house smoke free?
4. What do you think about social norm and culture to reduce exposure of passive smoking?
5. How peer support can help to reduce exposure of passive smoking?
6. Any other aspects to reduce the exposure of passive smoking?
