# Supplementary material for: A harm reduction model for environmental tobacco smoke exposure among Bangladeshi rural household children: A modified Delphi technique approach
Source: PLoS One. 2023 Feb 16;18(2):e0276424. doi: 10.1371/journal.pone.0276424 (PMC9934442; doi:10.1371/journal.pone.0276424)
Supplement: S3 File — (PDF) [file pone.0276424.s003.pdf]

Questionnaire for Modified Delphi Method

Do you think passive smoking exposure to the children in the rural area can be reduced by the following activities? Please rate the following actions.

No	Questions	9	8	7	6	5	4	3	2	1
**A**	**Smoke-Free Household Rules**									
1	Specifying a place for smoking in the household where children usually do not go.									
2	Design a no smoking sign by a child of the house and put it on doorway.									
3	Using a calendar and mark it after every smoking inside the house and check at the end of every month.									
4	Taking help from those who already practice smoke-free rules.									
5	Developing habit to smoke before coming home.									
6	Change dress and wash hand before going to near a child after smoking.									
**B**	**Religious Belief**									
7	As all major religions prohibited smoking. So by practicing religious rules properly can reduce exposure.									
8	Religious leader should inform the local people about the effect of smoking and passive smoking.									
**C**	**Peer Support**									
9	Support from spouse, parents and other household member.									
10	Educating children about the impact of passive smoking exposure and how to deal with it.									
**D**	**Social Awareness**									
11	Engaging members of local social welfare club to disseminate the harmful effect and aware local people for passive smoking exposure.									
12	'Social movement' such as implementing a fine (eg. 30 BDT) for smoking inside the house by local social welfare club which will be monitored by non-smoking elderly people or spouse in the house. The amount will be used for child welfare in the locality.									
13	Increasing social awareness through seminar.									
14	Distributing leaflets, banners and stickers about passives smoking.									
15	Using signboard and poster on passive smoking in the local tea stalls for disseminating awareness as most of the people of the village gather there.									
16	Organizing friendly popular sports (eg. Cricket, football) with embedded massage of passive smoking awareness.									
**E**	**Social Norm and Culture**									
17	Asking the husband not to smoke inside the house is being a social norm and culture in the rural area for quite a while. Woman empowerment can be a significant way to break the norm in some extent. Woman social community support group can help to empower woman through different activities and help to understand the effect of passive smoking and the importance to avoid the exposure.									
18	Adapting social value eg. Care for younger people.									

Note: 9: Strongly agree --- 1: Strongly disagree
