# Supplementary material for: A harm reduction model for environmental tobacco smoke exposure among Bangladeshi rural household children: A modified Delphi technique approach
Source: PLoS One. 2023 Feb 16;18(2):e0276424. doi: 10.1371/journal.pone.0276424 (PMC9934442; doi:10.1371/journal.pone.0276424)
Supplement: Attachment — Submitted filename: Response to Reviewers.docx [file pone.0276424.s004.docx]

**Manuscript ID: PONE-D-21-24273**

**Title:** A Harm Reduction Model for Environmental Tobacco Smoke Exposure among Bangladeshi Rural Household Children: A Modified Delphi Technique Approach

Journal Requirements:

1. Please ensure that your manuscript meets PLOS ONE's style requirements, including those for file naming. The PLOS ONE style templates can be found at

https://journals.plos.org/plosone/s/file?id=wjVg/PLOSOne_formatting_sample_main_body.pdf and

https://journals.plos.org/plosone/s/file?id=ba62/PLOSOne_formatting_sample_title_authors_affiliations.pdf

***Response:*** *Corrected accordingly.*

2. Please include additional information regarding the survey or questionnaire used in the study and ensure that you have provided sufficient details that others could replicate the analyses. For instance, if you developed a questionnaire as part of this study and it is not under a copyright more restrictive than CC-BY, please include a copy, in both the original language and English, as Supporting Information.

***Response:*** *Included the Questionnaire.*

3. Thank you for stating the following financial disclosure:

"No"

At this time, please address the following queries:

a)	Please clarify the sources of funding (financial or material support) for your study. List the grants or organizations that supported your study, including funding received from your institution.

b)	State what role the funders took in the study. If the funders had no role in your study, please state: “The funders had no role in study design, data collection and analysis, decision to publish, or preparation of the manuscript.”

c)	If any authors received a salary from any of your funders, please state which authors and which funders.

d)	If you did not receive any funding for this study, please state: “The authors received no specific funding for this work.”

Please include your amended statements within your cover letter; we will change the online submission form on your behalf.

***Response:*** *The authors received no specific funding for this work.*

4. We note that you have indicated that data from this study are available upon request. PLOS only allows data to be available upon request if there are legal or ethical restrictions on sharing data publicly. For information on unacceptable data access restrictions, please see http://journals.plos.org/plosone/s/data-availability#loc-unacceptable-data-access-restrictions.

In your revised cover letter, please address the following prompts:

a) If there are ethical or legal restrictions on sharing a de-identified data set, please explain them in detail (e.g., data contain potentially identifying or sensitive patient information) and who has imposed them (e.g., an ethics committee). Please also provide contact information for a data access committee, ethics committee, or other institutional body to which data requests may be sent.

b) If there are no restrictions, please upload the minimal anonymized data set necessary to replicate your study findings as either Supporting Information files or to a stable, public repository and provide us with the relevant URLs, DOIs, or accession numbers. Please see http://www.bmj.com/content/340/bmj.c181.long for guidelines on how to de-identify and prepare clinical data for publication. For a list of acceptable repositories, please see http://journals.plos.org/plosone/s/data-availability#loc-recommended-repositories.

We will update your Data Availability statement on your behalf to reflect the information you provide.

***Response:****Data included in the the following DOI*

https://doi.org/10.5061/dryad.f7m0cfxz7

**Reviewer 1:**

**Reviewer Comment**

1.In the cross-sectional study, it was indicated an overwhelming participant was male (70.7%) majority were married (85.6%) and completed from university (38.5%). Please check the data about the participant completed university education.

***Response:*** *We are thankful to the honourable reviewer for his comments. It was 37.5%.*

2. It seems that the research finding can not support the research goal.

***Response:*** *Thank you for the comment. The research goal was to develop a harm reduction model by applying the modified Delphi technique, and finally, the model was developed in this research.*

3. The manuscript looks like a research report, rather than a academic paper.

***Response:*** *We apricated the honourable reviewer comments. As the research is from the first author's doctoral thesis, the detailing of the manuscript is extensive, which may looks like a report.*

4. The tables in the manuscript need to be format again.

***Response:*** *Done accordingly to the Plos One Guideline*

5. The language need polish.

***Response:*** *Done accordingly.*

**Reviewer 2:**

**Reviewer Comment:**

Page 6: under Phase 3: evaluation of the model; It is suggested to mention what modification was done to the Delphi technique for better clarification of the modification in the method.

***Response****: Thank you for the query. We have included the following information “ The modified Delphi technique is alike to the full Delphi in terms of technique. The major adjustment contains of beginning the procedure with a set of prudently selected items. These pre-selected items may be drawn from several sources. The key advantage of this modification to the Delphi is that it typically improves the initial round response rate and also provides a solid foundation in previously developed work”*

• Page 6: under Phase 3: evaluation of the model; Calculation of the consensus on Interquartile range (IQR) was not clarified enough and recommended to have a proper explanation on how IQR was calculated based on the response score and the reason for the cut point is set at less than/ equal to 1. (This point links the result table shown in Page 17).

***Response:*** *To calculate the strength of the consensus, Interquartile range (IQR) is used. We have used the reference of Persai D, Panda R, Kumar R, Mc Ewen A. A Delphi study for setting up tobacco research and practice network in India. Tobacco induced diseases. 2016;14:4-. and Lefkothea Giannarou EZ. Using Delphi technique to build consensus in practice. Int Journal of Business Science and Applied Management,. 2014;9(2).*

• Page 21: “The outcomes did not validate with cotinine estimation” which needs proper explanation to mention. The reason is that the Bangladeshi people have tobacco smoking and habit of betel chewing with tobacco which can be a mixed-use of tobacco and can bias the result with cotinine estimation due to the misclassification of tobacco use (inclusive of without smoke).

***Response:*** *Thank you for the suggestion. We have included the suggested portion.*

• In figure 2: Just want to clarify that one item was not listed in the figure. Only 12 items out of 13 consensus criteria were reflected with one point omitting “distributing leaflets, banners and stickers about ETS”. Is that point covered under “using print materials” or missed to mention?

***Response:*** *The honourable reviewer is absolutely right. It was included under distributing leaflets, banners and stickers about ETS.*

**Reviewer 3:**

1. The sample includes households of 6 villages in the Munshiganj district, randomly selected from 64 districts. Although I understand logistics constraints, a sample of villages drawn from different districts would have been a much more natural procedure and results could have been extended to the Bangladeshi population. Taking villages from one district leads to results that are instead much more limited. I think that the overall paper should be rephrased by assuming that the target population is the rural part of the Munshiganj district.

***Response:*** *We apricated the honourable reviewer's view and partially agreed with his comments. Obviously, it would be great to obtain data from various districts of Bangladesh. However, that was very time consuming and needed funding. As Bangladesh is not a big country, rural areas are almost the same everywhere. That’s why the rural area reflects the other plain area except for the three hilly areas we mentioned in the research.*

2. If I understood correctly (not clear from text, please clarify), the dependent variable in the logistic regressions of Table 2 is always smoke exposure, included as a self-reported binary variable. I'm quite a bit concerned about the dichotomous definition of exposure: what do 0 and 1 exactly mean here? How can we be sure that all the subjects give to 0 and 1 the same meaning? Without a clear-cut definition of the dependent variable, the results of table 2 are difficult to interpret.

***Response:*** *In this research**, exposure to passive smoking indicates exposure to another person’s tobacco smoke in the household for at least 15 minutes daily for more than one day every week in the past 30 days. Though we have collected the data through a self-administered questionnaire, the researchers gave a brief description of how to fill the data, and if they failed to understand, they were provided with a toll-free number to discuss with the researcher.*

3. Table 2 displays the results of a battery of logistic regressions, where some relevant covariates are included separately and then adjusted for confounders. This is not a standard approach: relevant covariates should be included simultaneously. I'd welcome a single, carefully selected (see major issue 4 below) logistic regression that includes the ORs of all the relevant covariates and the confounders.

***Response:*** *Thank you for the comments. The adjusted portion of the table was not included separately. It was included together. However, only the significant outcome was shown in the table. For better understanding, we include the full table here.*

**Logistic regression: Odds ratios (ORs) and 95% confidence intervals (CI) of demographic variables and independents variables with dependent variable**

| Variable | B | Sig. | OR | 95% CI for OR  Lower Upper | |
| --- | --- | --- | --- | --- | --- |
| Variables which are significant in the regression model | | | | | |
| Smoker in the household | | | | | |
| Yes | Reference |  |  |  |  |
| No | -5.146 | .000* | .006 | .002 | .021 |
| Household smoking rules | | | | | |
| No/low implementation | Reference |  |  |  |  |
| Highly implementation | -5.222 | .000* | .005 | .001 | .058 |
| Social norm and culture | | | | | |
| Low influence | Reference |  |  |  |  |
| Moderate influence | -3.094 | .009* | .045 | .004 | .461 |
| High influence | -3.771 | .001* | .023 | .002 | .224 |
| Subjective Norm | | | | | |
| Negative | Reference |  |  |  |  |
| Neutral | -3.734 | .017* | .024 | .001 | .510 |
| Positive | -3.548 | .019* | .029 | .001 | .561 |
| Variables which are not significant in the regression model | | | | | |
| Gender | | | | | |
| Male | Reference |  |  |  |  |
| Female | -.115 | .885 | .891 | .189 | 4.210 |
| Age in year | | | | | |
| 18-24 | Reference |  |  |  |  |
| 25-44 | -1.182 | .152 | .307 | .061 | 1.545 |
| 45-64 | .034 | .973 | 1.034 | .153 | 6.977 |
| 65+ | 1.339 | .506 | 3.816 | .074 | 197.624 |
| Marital status | | | | | |
| Single | Reference |  |  |  |  |
| Married | -.039 | .948 | .961 | .292 | 3.162 |
| Education | | | | | |
| No formal schooling | Reference |  |  |  |  |
| Primary schooling | .653 | .620 | 1.921 | .146 | 25.259 |
| Secondary schooling | .536 | .693 | 1.709 | .119 | 24.493 |
| College | -.482 | .743 | .618 | .035 | 11.008 |
| University | -.668 | .650 | .512 | .028 | 9.223 |
| Employment | | | | | |
| Unemployed | Reference |  |  |  |  |
| Service holder | -.415 | .622 | .661 | .127 | 3.434 |
| Business | .191 | .837 | 1.210 | .197 | 7.420 |
| Agriculture | .789 | .372 | 2.201 | .389 | 12.448 |
| Religion | | | | | |
| Islam | Reference |  |  |  |  |
| Other | 1.083 | .156 | 2.953 | .661 | 13.203 |
| Household income in BDT | | | | | |
| <5000 | Reference |  |  |  |  |
| 5000-10000 | .114 | .899 | 1.121 | .193 | 6.495 |
| 10001-15000 | .916 | .300 | 2.500 | .442 | 14.127 |
| 15001-20000 | -.938 | .325 | .391 | .061 | 2.532 |
| 20001-30000 | -1.185 | .229 | .306 | .044 | 2.108 |
| >30000 | 1.466 | .197 | 4.330 | .467 | 40.167 |
| Number of children in the household | | | | | |
| 1 | Reference |  |  |  |  |
| 2-3 | .632 | .306 | 1.882 | .561 | 6.313 |
| 4-5 | -1.524 | .100 | .218 | .036 | 1.336 |
| 6-7 | 1.728 | .834 | 5.632 | .000 | 56707685.132 |
| Media | | | | | |
| Low | Reference |  |  |  |  |
| High | -1.949 | .277 | .142 | .004 | 4.799 |
| Household and Environment | | | | | |
| Low | Reference |  |  |  |  |
| High | -.125 | .813 | .883 | .314 | 2.478 |
| Knowledge | | | | | |
| Low | Reference |  |  |  |  |
| Moderate | -.894 | .113 | .409 | .135 | 1.236 |
| High | -.186 | .796 | .830 | .203 | 3.399 |
| Self-belief | | | | | |
| Low | Reference |  |  |  |  |
| High | 4.391 | .250 | 80.689 | .045 | 144177.473 |
| Religious belief | | | | | |
| Low | Reference |  |  |  |  |
| Moderate | 1.256 | .326 | 3.511 | .287 | 43.026 |
| High | .130 | .902 | 1.139 | .143 | 9.060 |
| Social awareness | | | | | |
| Low | Reference |  |  |  |  |
| High | .493 | .688 | 1.638 | .148 | 18.170 |
| Intention | | | | | |
| Negative | Reference |  |  |  |  |
| Neutral | 1.321 | .725 | 3.749 | .002 | 5903.248 |
| Positive | -1.593 | .183 | .203 | .020 | 2.119 |
| Attitude | | | | | |
| Negative and Neutral | Reference |  |  |  |  |
| Positive | -2.359 | .087 | .094 | .006 | 1.410 |
| Perceived behavior control | | | | | |
| Negative | Reference |  |  |  |  |
| Neutral | -.683 | .490 | .505 | .073 | 3.512 |
| Positive | -.028 | .977 | .972 | .142 | 6.641 |

4. Model checking is overlooked. Without asking for a full model diagnostic, the authors should at least provide some residual analysis that show the goodness of fit of the model.

***Response:*** *Thanks for the comments. The model was checked.* The model predicted 91.7% of the correct estimate. The model chi-square (x2=371.792, d.f. = 40, p = 0.000), assessing goodness-of-fit, was significant, indicating that the independent variables are not related to the log odds of the dependent variable. The Hosmer and Lemeshow test was not significant (x2=7.481, d.f. = 8, p = 0.486), confirming the model's goodness of fit.

6. Although the English is generally correct, some sentences should be revised. Examples are: “The cross-sectional study observed”, “Through purposive sampling, the experts were selected”, “completed from university”, “It's not liked the town”…

***Response:*** *Thank you. We have tried to correct the sentence.*

7. The caption of Table 2 remarks that the ORs are adjusted for some confounders (age, gender, income, religious belief, knowledge, environmental impact, media influence, perception, attitude, intention, perceived behavior control). Only some of these variables are summarized by Table 1: what about the others? In addition, Table 1 should display the ORs associated with these confounders, too.

***Response:*** *Thank you for the query. Kindly Check the response of question no 3.*
