# Supplementary material for: A harm reduction model for environmental tobacco smoke exposure among Bangladeshi rural household children: A modified Delphi technique approach
Source: PLoS One. 2023 Feb 16;18(2):e0276424. doi: 10.1371/journal.pone.0276424 (PMC9934442; doi:10.1371/journal.pone.0276424)
Supplement: Attachment — Submitted filename: Response to Reviewers.docx [file pone.0276424.s005.docx]

**Manuscript ID: PONE-D-21-24273**

**Title:** A Harm Reduction Model for Environmental Tobacco Smoke Exposure among Bangladeshi Rural Household Children: A Modified Delphi Technique Approach

Journal Requirements:

1. Please review your reference list to ensure that it is complete and correct. If you have cited papers that have been retracted, please include the rationale for doing so in the manuscript text, or remove these references and replace them with relevant current references. Any changes to the reference list should be mentioned in the rebuttal letter that accompanies your revised manuscript. If you need to cite a retracted article, indicate the article’s retracted status in the References list and also include a citation and full reference for the retraction notice.

***Response:*** *Corrected accordingly.*

*Please check reference numbers 6, 7*

**Reviewer 3:**

**Reviewer Comment:**

1. I still think that taking villages from one district coud lead to results that are difficult to extend to the general population. Although the authors disagree on this point, I would suggest to add this issue among the possbile limitations of the study

***Response****: Thank you for the suggestion. I have added the limitation on page 22 lines 355-356.*

2. Lines 245-248: "The model predicted 91.7% of the correct estimate. The model chi-square (x2=371.792, df. = 40, p = 0.000), assessing goodness-of-fit, was significant, indicating that the independent variables are not related to the log odds of the dependent variable." The meaning of these two sentences is not clear. I suggest to remove them. The Hosmer and Lemeshow test provides enough information about the model goodness of fit.

***Response:*** *According to the advice of the reviewer, the lines were removed.*

3) Data: I was not able to retrieve the data from Dryad, using the address the authors provided, doi:10.5061/dryad.f7m0cfxz7. Please provide a link that directly points to the dataset

***Response:*** *Thank you for the query. Please check* [*https://datadryad.org/stash/share/kNWzQQ4egnoLlymWwJ2xmOPFi0mS0qIh9lghKdZFmsE*](https://datadryad.org/stash/share/kNWzQQ4egnoLlymWwJ2xmOPFi0mS0qIh9lghKdZFmsE)
